# Vitamin A Status in Cystic Fibrosis in the Current Era of CFTR-Directed Therapies

**DOI:** 10.3390/nu18162639

**Published:** 2026-08-12

**Authors:** Senthilkumar Sankararaman, Terri Schindler, Kay Vavrina, Maria Mascarenhas

**Affiliations:** 1 Division of Pediatric Gastroenterology, Cleveland Clinic Children’s Hospital, Cleveland, OH 44195, USA; 2 Division of Pediatric Pulmonology, UH Rainbow Babies & Children’s Hospital, Cleveland, OH 44106, USA; terri.schindler@uhhospitals.org; 3 Gastroenterology Care, AbbVie, 7575 Frankford Road #3213, Dallas, TX 75252, USA; kay.vavrina@abbvie.com; 4 Division of Gastroenterology, Hepatology, and Nutrition, Children’s Hospital of Philadelphia, Philadelphia, PA 19104, USA; mascarenhas@chop.edu

**Keywords:** CF, retinol, beta carotene, xerophthalmia

## Abstract

Vitamin A plays an important role in multiple homeostatic functions such as vision, immunity, epithelial cell integrity and differentiation, cell signaling, pulmonary function, reproduction, growth, and development. Vitamin A deficiency was described in people with cystic fibrosis (pwCF) due to a multitude of causes, such as suboptimally managed exocrine pancreatic insufficiency, advanced cystic fibrosis-related liver disease (aCFLD), and a history of intestinal resection, and is generally rare in contemporary practice. Apart from true vitamin A deficiency, low vitamin A levels may also be noted in various inflammatory states, as vitamin A is a negative acute-phase reactant. In the current era of cystic fibrosis (CF) transmembrane-conductance regulator (CFTR)-directed therapies, there is a paradigm shift in vitamin A status, with deficiency statuses becoming rarer, and instead higher serum vitamin levels (in some cases, even in the hypervitaminosis range) are increasingly reported. People with aCFLD, renal insufficiency, post-lung transplantation, and pregnancy are prone to vitamin A toxicity. Hence, CF clinicians should be proactive in evaluating these abnormalities and proficient in managing both deficiency and toxicity, as both these conditions can be associated with adverse outcomes. In this review, we detailed the basics of vitamin A metabolism, manifestations of both vitamin A deficiency and excess, and their clinical implications in pwCF.

## 1. Introduction

**Figure 1:**
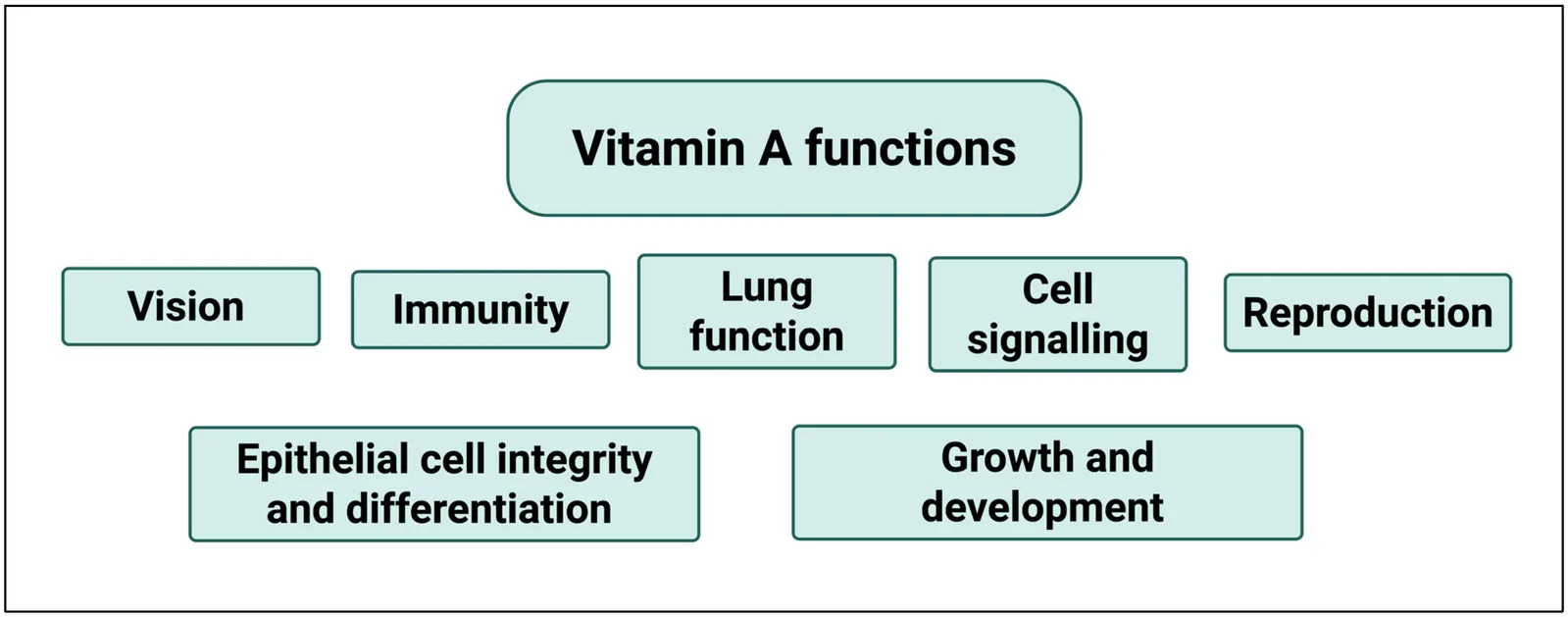
Salient functions of vitamin A. Created in BioRender. Sankararaman, S. (2026). https://BioRender.com/di49h5r.

Vitamin A plays an indispensable role and is vital for vision, immunity, epithelial cell integrity and differentiation, cell signaling, pulmonary function, reproduction, growth, and development (Figure 1) [1,2,3,4]. People with cystic fibrosis (pwCF) are at increased risk of vitamin A deficiency. In fact, Dr Dorothy Andersen’s first description of cystic fibrosis (CF) in 1938 reported vitamin A deficiency in 20% of the cases [5]. Since then, many investigators have documented vitamin A deficiencies in pwCF [6,7,8,9,10,11,12,13,14,15,16].

Exocrine pancreatic insufficiency (EPI), noted in approximately 85% of pwCF, is one of the major reasons for vitamin A malabsorption along with malabsorption of fat and other fat-soluble vitamins (vitamins D, E, and K) [3,17,18]. Additionally, several reasons could predispose pwCF to vitamin A deficiency. Bile salts play an indispensable role in the absorption of fats and fat-soluble vitamins through the formation of micelles [19]. The cystic fibrosis transmembrane-conductance regulator (CFTR) channel dysfunction in the hepatobiliary system and gastrointestinal tract could lead to impaired bile secretion, defective micelle formation, and abnormal acidic mucus, further exacerbating fat and fat-soluble vitamin malabsorption [20,21]. Low vitamin A status in CF has been associated with poor clinical status, worse pulmonary function, and increased pulmonary exacerbations [22,23,24]. The majority of studies involving vitamin A in pwCF include either retrospective studies or single-center observational trials [3,25]. In the absence of robust evidence, most recommendations in vitamin A management in CF come from expert consensus and practice guidelines [4,26,27,28,29,30].

In the current era of CFTR-directed therapies, there is a paradigm shift in vitamin A status, with deficiency statuses becoming rarer, and instead higher serum vitamin levels (in some cases, even in the hypervitaminosis range) are increasingly reported [31,32]. Hence, clinicians who manage CF should be proficient in managing both deficiency and toxicity, as both these conditions can be associated with adverse outcomes. Further, highlighting this wide spectrum of vitamin A status, ranging from deficiency to hypervitaminosis, in the era of CFTR-directed therapies underscores the critical need for personalized and individualized nutritional management.

In this article, we detailed the basics of vitamin A metabolism (including its pharmacokinetics and pharmacodynamics), manifestations of both vitamin A deficiency and excess, and their clinical implications in pwCF.

## 2. Fundamentals of Vitamin A

Humans cannot produce vitamin A and hence must obtain it exclusively through diet, either as preformed vitamin A (retinol or retinyl esters) or as provitamin A compounds, namely α-carotenoids, β-carotenoids, and β-cryptoxanthin, and among these, β-carotenoids are the most important [33]. Vitamin A collectively represents all-*trans*-retinol, retinyl ester (storage form of retinol), and its active metabolites, 11-*cis*-retinal (aldehyde-form), and all-*trans*-retinoic acid (acid-form) [34]. In this article, the terms retinol and vitamin A are used interchangeably. Retinal plays an indispensable role in dark adaptation and participates in the rhodopsin visual cycle [34,35]. Rods are more vulnerable to vitamin A deficiency than cones [36]. Retinoic acid acts as a messenger involved in cell differentiation and is a ligand for nuclear receptors, called retinoic acid receptors/retinoid X receptors, and modulates transcription of several (>500) genes in cell growth and differentiation, embryogenesis, reproduction, and mucosal immunity [34,35,37]. Further, β-carotene also plays a vital role in the antioxidant defense system along with vitamins C, E, and selenium. Hence, deficiency of vitamin A can lead to ophthalmologic manifestations, skin disorders, immune dysfunction, and poor growth [7,13].

### 2.1. Sources of Vitamin A and β-Carotenoids

Vitamin A is ingested as either retinol or β-carotenoids. Retinol is prevalent in animal sources such as liver, eggs, and dairy products. Provitamin A carotenoids are found in yellow-colored vegetables and fruits such as carrots, sweet potatoes, apricots, cantaloupes, peaches, squash, papaya, and pumpkin, and also in dark green leafy vegetables, such as spinach, kale, collards, and broccoli [38]. Other common sources of vitamin A and its derivatives include oral/enteral nutritional supplements (beverages), multivitamins including CF-specific vitamins (Supplementary Table S1), over-the-counter vitamin products, and medications of vitamin A derivatives called retinoids (e.g., isotretinoin and acitretin, which are often used in inflammatory dermatological disorders) [39].

Because vitamin A can be consumed as either retinol or β-carotene, retinol activity equivalent (RAE) is defined to standardize comparisons across various sources of vitamin A. One μg RAE is equivalent to one μg retinol (which is 3.33 international units (IU)) or 12 μg β-carotene [38,40,41]. The tolerable upper intake level (UL) for retinol is 3000 μg (10,000 IU) for the general adult population. Carotenoids do not have a UL as they have lower gastrointestinal absorption compared to retinol, and more importantly, due to homeostatic regulation (detailed in the next section) of their conversion to retinol [34].

### 2.2. Gastrointestinal Absorption, Distribution, Storage, and Metabolism

#### 2.2.1. Gastrointestinal Absorption

**Figure 2:**
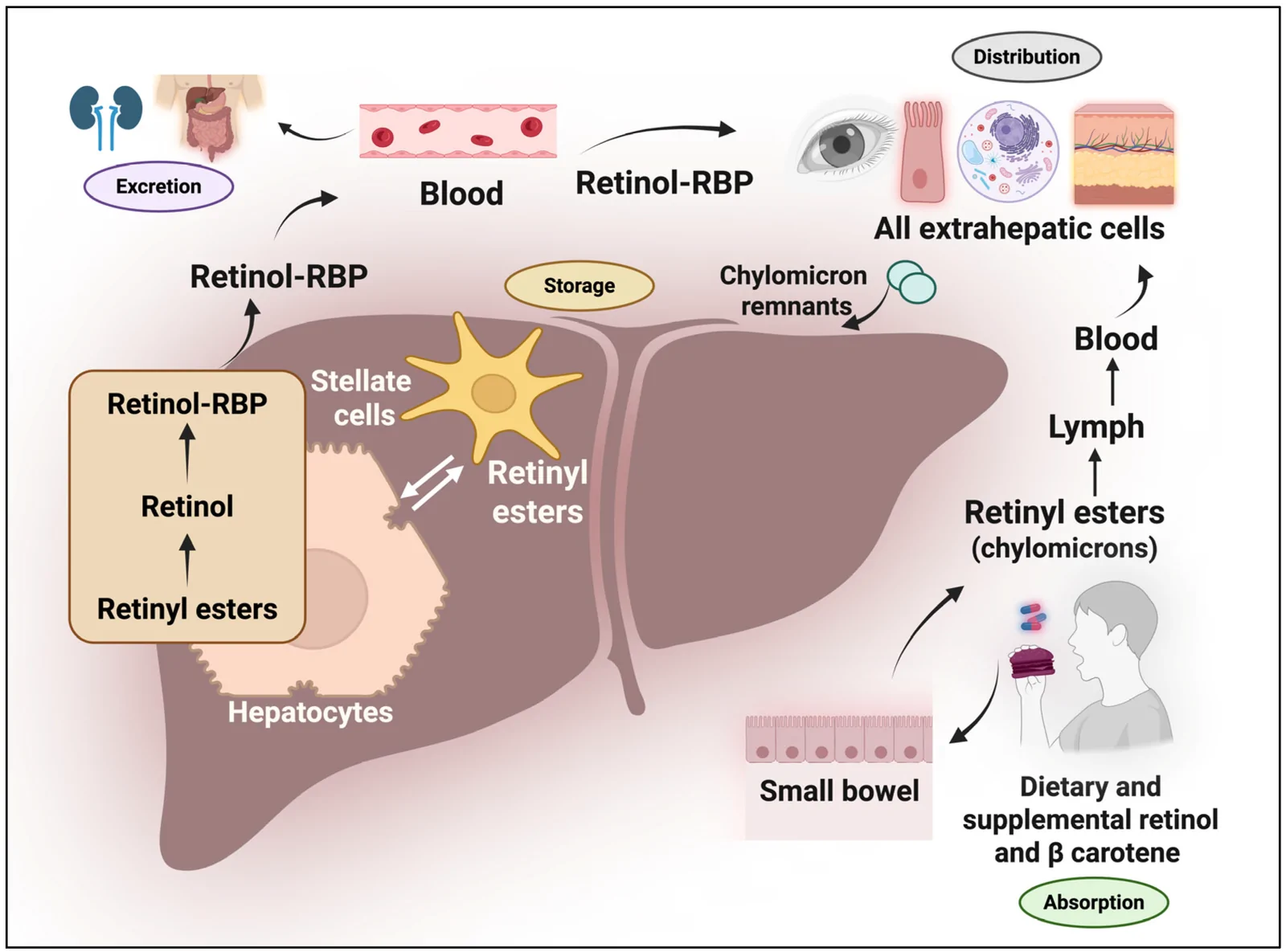
Gastrointestinal absorption, storage, peripheral distribution, and metabolism of Vitamin A. Created in BioRender. Sankararaman, S. (2026) https://BioRender.com/c6bmi35.

Almost 70–90% of preformed vitamin A is absorbed in the gastrointestinal tract [42]. Retinyl esters (mainly as retinyl palmitate) and carotenoids are lipid-soluble and therefore lipase and dietary lipids aid in their absorption [2]. Retinol and β-carotene have differences in gastrointestinal absorption and metabolism. Retinyl esters undergo hydrolysis and, along with lipids and bile salts, form micelles that are absorbed by enterocytes [2,35]. The retinol is then esterified back to retinyl esters (mainly as palmitate), and some uncleaved carotenoids are incorporated in chylomicrons, which travel via the lymphatics into the bloodstream (Figure 2) [19,43].

On the contrary, provitamin carotenoids do not need hydrolysis and enter via facilitated absorption in the small intestine. In the enterocytes, most provitamin A carotenoids are oxidatively cleaved to retinol by the enzyme β-carotene 15,15′-dioxygenase [33,35,44,45]. The metabolism of β-carotenoids into active vitamin A is a highly regulated step that is subject to feedback regulation, depending on bodily vitamin A status. A fraction of β-carotenoids is absorbed without being converted into retinol, and this is also subject to regulatory absorption [46,47]. This conversion of β-carotene to vitamin A may be influenced by the existing vitamin A status and also, to some extent, dependent on the dose ingested [34]. However, the exact details of this homeostatic conversion to retinol remain elusive. Unlike retinol, excessive consumption of β-carotene causes a reversible, harmless condition called carotenemia with yellowish-orange discoloration of the skin.

#### 2.2.2. Distribution and Storage of Vitamin A

In the systemic circulation, retinyl esters and carotenoids are utilized by the peripheral tissues, but most of them reach the liver as chylomicron remnants for storage [19,35]. Approximately 80% of vitamin A is stored in the perisinusoidal stellate cells (also known as Ito cells) of the liver as retinyl esters (mainly as palmitate) in fat droplets of the cytoplasm [48,49,50]. Stellate cells also have a key role in hepatic uptake and release of retinoids [49].

From the liver, retinol is transported to peripheral tissues bound to retinol-binding protein-4 (RBP-4). RBP-4 is produced by the hepatocytes and is the major transport protein for retinol [19,42,48,51]. RBP-4 is one of the retinol-binding proteins (RBPs) and will be simply referred to as RBP in this review [52]. Despite variable daily dietary and supplemental vitamin A ingestion, a steady state of serum retinol is maintained by timely secretion of retinol bound to apo-RBP (protein moiety) to form holo-RBP (retinol-RBP) in a 1:1 molar ratio [43,53]. Approximately 95% of the serum RBP is coupled with another protein, transthyretin (TTR, which is also referred to as the prealbumin and is produced by the liver) in a ratio of 1:1 [52,54,55]. The binding of retinol-RBP to TTR reduces the glomerular filtration of retinol, as this complex is too large to pass through [54,55]. It should be noted that serum retinol is homeostatically maintained in the serum across a wide range of total body stores, ranging from deficiency to excess status [34]. The hepatic storage of retinyl palmitate in the general population usually lasts for almost 1–2 years under normal circumstances [53,56]. When liver stores are severely depleted, serum retinol concentrations may then begin to fall into a suboptimal range.

Circulating retinol is taken up by target cells via the receptor STRA6 [48]. In the eyes, retinol is oxidized to retinaldehyde (retinal) [48]. This oxidative conversion of retinol to retinal is reversible and requires the action of a zinc-dependent retinol dehydrogenase enzyme [57]. In all other tissues, retinal is further oxidized to all-*trans*-retinoic acid, and this step is irreversible. As noted earlier, retinoic acid, the active form of vitamin A, participates in the transcription of several genes involved in essential functions such as cell growth, organogenesis, immune function, etc. [34,35,37].

#### 2.2.3. Excretion of Vitamin A

The clearance of retinoic acid is mediated predominantly by the cytochrome P450 family 26 (CYP26) enzyme system in the liver and kidneys to glucuronides via bile and urine, respectively [58]. The kidneys also play an important role in retinol homeostasis. In the glomeruli, the filtered retinol-RBP complex is reabsorbed in the proximal tubule cells by the endocytic receptor protein, megalin [48]. When kidney function declines with reduced glomerular filtration, the excretion of retinoids and RBP may decrease, resulting in increased accumulation of vitamin A [59].

## 3. Measurement of Vitamin A Status

### 3.1. Serum Retinol and Retinol-Binding Protein

Estimation of vitamin A concentration in the liver is the gold standard way to define vitamin A status, and a level of <0.10 μmol/g of liver tissue is generally considered as vitamin A deficiency [60]. However, this method is invasive, impractical, and obsolete in clinical practice. In clinical circumstances, serum all-*trans*-retinol is the most commonly used biomarker for estimating vitamin A deficiency. Investigators have noted serum vitamin A correlated well with its liver stores in pwCF and hence can be used as a clinical surrogate for estimating vitamin A status [61].

Estimation of vitamin A is recommended at CF diagnosis and thereafter annually or 3–6 months after a dosage change [29]. A serum retinol cut-off of ≤0.20 mg/L (or 20 μg/dL or 0.70 μmol/L) is generally indicative of vitamin A deficiency status [62,63]. A level ≤ 0.10 mg/L indicates severe deficiency. The cut-off range varies with age, and clinicians should follow the reference range recommended by the laboratory [64,65,66,67]. Most CF centers use reference laboratories, and the specimen should be light-protected immediately after procurement [66,67]. Many laboratories use liquid chromatography tandem mass spectrometry for vitamin A estimation [66,67]. Serum retinol levels generally correlate very well with the prevalence and severity of vitamin A deficiency manifestations (night blindness and xerophthalmia), and levels improve in response to treatment interventions [35].

A serum retinol cut-off of ≤0.70 μmol and RBP cut-off of ≤0.70 μmol/L predicted 87% sensitivity and 98% specificity for detecting vitamin A deficiency status [62]. RBP levels demonstrate excellent correlation with serum retinol, and measurement of serum RBP along with vitamin A is recommended [53]. A molar ratio of retinol/RBP < 0.8 is considered indicative of vitamin A deficiency [20,68,69]. Here, clinicians should ensure that both retinol and RBP are in the same units. The conversion equation for retinol (µg/dL) × 0.0349 = retinol value in µmol/L and for RBP(mg/dL) × 0.476 = RBP in µmol/L [20,69,70].

There are several caveats clinicians should be aware of when evaluating vitamin A status. Estimation of serum retinol and RBP should be avoided when patients have acute pulmonary exacerbation or other infections/inflammatory states, as both serum retinol and RBP are negative acute-phase reactants and can be erroneously low in infection/inflammatory states, resulting in a reduction in the hepatic synthesis of RBP [34,53,65,71,72]. Retinol estimation should ideally be done during clinical stability as part of annual outpatient laboratory measurements [25]. Some experts also advocate concomitant routine measurement of C-reactive protein (CRP), with serum retinol estimation, to assess subclinical infection/inflammatory status [73]. In a study, nearly one-fourth of patients had low vitamin A levels in the deficiency range, and lower vitamin A levels correlated with high levels of CRP [72]. In another study, serum retinol was negatively associated with higher serum IgG levels (used as a surrogate for inflammation) [74]. In both studies, decreased serum retinol levels were indicative of suppression related to inflammation and/or infection rather than true vitamin A deficiency [72,74]. Levels are ideally assessed in the fasting state, as nonfasting levels may confound recent intake of vitamin A [20].

Retinol levels are a better indicator of deficiency than of excessive status [35,75,76,77]. There is no defined level for vitamin A toxicity. However, for the upper limit of the normal range, the population-based 95th percentile of the National Health and Nutrition Examination Survey (NHANES) includes 0.72 mg/L (or 72 μg/dL or 2.5 μmol/L), which is often referred to as the upper limit of reference [78]. For evaluation of vitamin A excess state, the proportion of retinyl esters in serum retinol (normally, <10% of total levels) is the preferred test [35,75,76,77]. A molar ratio of retinol/RBP > 1.2 (some advocate a ratio of >1) also represents vitamin A excess status [20,68,69].

### 3.2. Plasma Zinc

Zinc participates in crucial aspects of vitamin A metabolism, including its absorption, transport, and utilization [57,79]. Zinc deficiency decreases serum retinol concentrations because of its role in the hepatic synthesis or secretion of RBP, even in the presence of adequate liver vitamin A stores [57]. The regulatory role of zinc is also mediated through the oxidative conversion of retinol to retinal, which requires the action of a zinc-dependent retinol dehydrogenase enzyme [57]. Evaluation of zinc deficiency should be considered in patients with refractory vitamin A deficiency [27,80]. However, routine additional zinc supplementation may not affect plasma levels of vitamin A and RBP [81]. The important laboratory parameters used to assess both vitamin A deficiency and excess status were summarized in Table 1. Estrogens, either endogenous or those used in contraceptive agents or hormone replacement therapy, may increase serum retinol and RBP due to their increased mobilization from the liver [57].

## 4. Clinical Manifestations of Vitamin A Deficiency

Night blindness or nyctalopia (characterized by abnormal dark adaptation) is the most common and earliest manifestation of vitamin A deficiency. Rods are more sensitive to vitamin A deficiency than cones; conjunctival xerosis (xerophthalmia) and Bitot spots occur later in vitamin A deficiency [6,9,12,19,25,34,82,83,84,85]. The appearance of the Bitot spot has been described as “ripples in the sand at receding tide” [12]. In advanced stages, loss of vision (keratomalacia), skin changes (follicular hyperkeratosis), and recurrent infections are noted [7,8,86,87]. The dark adaptation test is commonly administered for evaluating night blindness and can also be used to monitor the effect of vitamin A therapy. Electroretinogram may show a reduction in scotopic responses in vitamin A deficiency [19,80].

## 5. Manifestations of Hypervitaminosis A

Symptoms of hypervitaminosis A in early stages include non-specific manifestations such as decreased appetite, headache, nausea, and dry skin [41,59]. In later stages, increased intracranial tension, such as irritability, papilledema, and visual disturbances, can be noted. Also, liver toxicity and bone demineralization resulting in pain and hypercalcemia may occur in chronic hypervitaminosis A [88,89]. Concomitant use of medications such as tetracyclines with vitamin A may increase the risk of raised intracranial tension, and a consulting pharmacist may help prevent these iatrogenic complications [20,90]. Clinicians should have a high index of suspicion for conditions linked to vitamin A toxicity, such as raised intracranial pressure, in pwCF on elexacaftor/tezacaftor/ivacaftor (ETI) (detailed in a later section). Regular screening for symptoms such as headache, nausea, vomiting, and visual disturbances is essential. Based on clinical suspicion, fundoscopy to assess for papilledema may be urgently warranted.

## 6. Clinical Scenarios Where Vitamin A Status Can Be Disrupted

**Figure 3:**
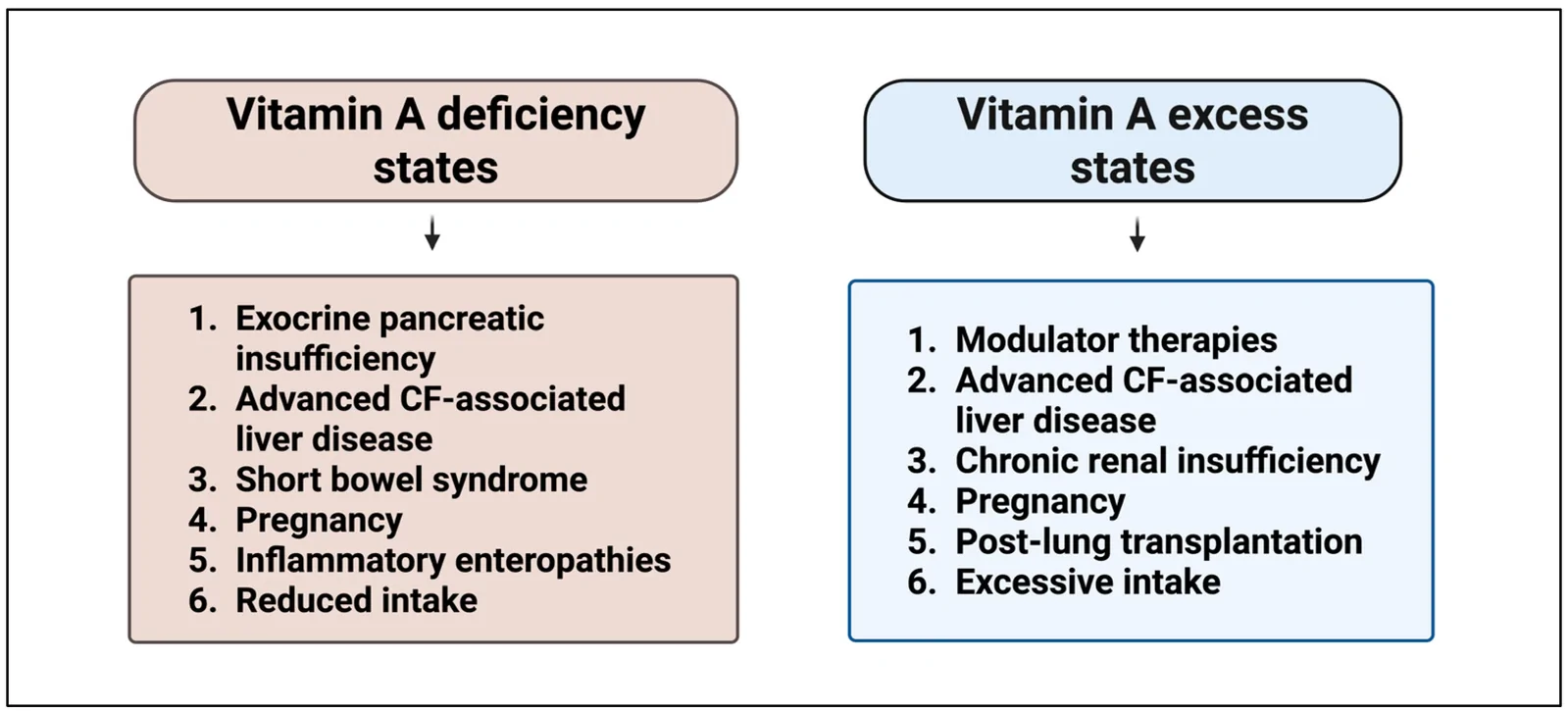
Vitamin A deficiency and excess states in cystic fibrosis. Created in BioRender. Sankararaman, S. (2026). https://BioRender.com/ehapnq1.

In pwCF, both deficiency and excess states can be noted in many circumstances (Figure 3). A summary of these conditions was included in Table 2.

### 6.1. PwCF on CFTR Modulators

#### 6.1.1. Vitamin A Status Before Widespread Use of the Modulators

Vitamin A deficiency in the pre-modulator era has been reported in 2–40% of pwCF [6,16,18,74,78,103,129,130,131,132,133]. The prevalence of vitamin A deficiency has been shown to be even higher at birth (50%), which improves following pancreatic enzyme replacement therapy and vitamin A supplementation [2,6,134]. Recent studies demonstrated that vitamin A deficiency in CF has become less frequent overall [135,136]. However, there are specific instances where both deficiency and excess still occur, and vitamin A status needs to be carefully monitored. Vitamin A deficiency remains common in suboptimally controlled EPI and people with short bowel syndrome (SBS) [16,19,24,92].

Increased vitamin A levels (without manifestations of toxicity) have been noted in rare instances even in the pre-ETI era, likely due to increased dietary or supplemental sources (specifically water-miscible retinol supplements) [7,25,77,137]. Vitamin A toxicity generally occurs when intakes exceed >10,000 μg RAE for an extended period of time or repeated high doses (e.g., 100,000–200,000 IU) over a short period of time. Woestenenk and colleagues noted that total vitamin A intake exceeded the UL in nearly one-third of 862 dietary assessments involving 221 children with CF [74]. The higher intake was noted predominantly in children less than 6 years of age [74]. Estimating serum retinyl esters (the storage form) can be more useful for estimating vitamin A toxicity, and >10% of total vitamin A in the form of retinyl esters is considered abnormal [60,77]. As RBP is produced by the liver, its level may be lower in people with aCFLD. A molar ratio of retinol/RBP > 1.2 (some advocate a ratio of >1) represents vitamin A excess status [20,68,69].

Even in the pre-modulator era, researchers have found that approximately 20% to nearly 50% of pwCF with EPI had retinol levels above the recommended range [78,138,139]. In non-CF studies, excessive vitamin A levels were also associated with worsening outcomes, and this entity is termed “the vitamin A paradox” [140]. In the excess state, vitamin A can cause toxicity to the liver and skeletal systems, and may also interfere with vitamin D metabolism [77,141]. Frequent vitamin A levels are recommended to monitor for toxicity when patients consume vitamin A-containing medications such as isotretinoin [20,90].

#### 6.1.2. Evidence Regarding Vitamin A Status After Modulator Initiation

Ivacaftor, a potentiator, alone is indicated for gating variants (e.g., G551D), while combination regimens (ivacaftor with correctors) are used for pwCF carrying at least one F508del allele. Higher serum vitamin A levels, mostly in the non-toxic range, have been noted after the initiation of modulators [103,104,105,106,107,108,109,110,111,112,113,116,142,143]. These studies varied in methodology, sample size, demographics (age, genotype), clinical characteristics (modulator used, pancreatic status, presence of liver disease, duration of follow-up, etc.), and despite that, the increase in vitamin A has been consistently noted. Many of these studies evaluated the changes in vitamin D and vitamin E levels after modulator initiation. Unlike vitamin A, the changes in vitamin D and vitamin E levels were not consistent after initiation of modulators.

Initially, ivacaftor was noted to increase serum vitamin A levels [143]. Lumacaftor–ivacaftor and tezacaftor/ivacaftor also significantly increased retinol levels [114,142]. In a study by Francalanci et al., vitamin A levels were evaluated after modulators, and levels were higher after ivacaftor, lumacaftor–ivacaftor, and elexacaftor/tezacaftor/ivacaftor (ETI) [103]. While the increase in retinol was not statistically significant post ivacaftor, the increase was significant after lumacaftor–ivacaftor and ETI [103]. With ETI, several studies noted an increase in serum vitamin A levels [103,104,105,106,107,108,109,110,111,112,113,114,115,116].

In a pediatric study, Schembri et al. noted a significant increase in vitamin A levels in three participants following ETI initiation [107]. Here, one child had elevated levels in just 24 days after ETI. In another study (presented in abstract form), the proportion of pwCF after modulator therapy (unclear which modulators were evaluated) with raised vitamin A levels increased significantly from 3% in 2019 to 18% by 2023 (*p* < 0.001) [92]. Alicandro and colleagues noted that the mean retinol level increased by 0.11 mg/L during one year of follow-up, but at the end of two years, retinol returned closer to baseline levels [116]. On the contrary, other studies did not note a significant increase in vitamin A immediately after initiation of ETI but noted a significant increase at one year [111].

Investigators also noted that individuals with high vitamin A generally had higher median body mass index and forced expiratory volume in 1 s (FEV_1_) compared to those with low vitamin A status, reiterating the concept that higher vitamin A levels may be more likely in less sick patients [144,145]. In both referenced studies, most pwCF had EPI (89–92%), and the vitamin A levels correlated with FEV_1_ even after adjusting the data for pancreatic status.

In a large prospective study involving 14 CF units in Spain, 224 patients with a median age of 14 years (IQR—11.4 to 16.3) were recruited [146]. The majority of patients (90.6%) had EPI. Fifty-one patients received dual modulators (either lumacaftor–ivacaftor or tezacaftor/ivacaftor and 173 had ETI. Vitamin levels were evaluated at baseline (before modulator use) and at 6 and 12 months post-treatment. In the overall population, vitamin A levels were higher at both 6 and 12 months when compared to baseline values. Overall, vitamin A increased from baseline to 6 months and from baseline to 12 months. In the ETI group, the increase was higher than in the lumacaftor–ivacaftor or tezacaftor/ivacaftor group. Moreover, this increase was maintained at 12 months in the ETI group, while in the dual modulator group, the increase was not sustained at 12 months. In the ETI group, a decrease in the proportion of deficient vitamin A values (<30 mcg/dL) from baseline at 6 months (21% vs. 6%, *p* = 0.0034). As an additional finding, it should be noted that the ETI group showed an increase in vitamin A toxic levels at 6 months compared to baseline (3% vs. 11%, *p* = 0.0117) [146]. In another study, the significant increase in vitamin A was sustained at 24 months post ETI [143].

While many studies noted an increase in vitamin A within clinically acceptable ranges, case reports of fulminant intracranial hypertension due to hypervitaminosis have been reported following ETI initiation [31,147]. Intracranial hypertension is a known adverse effect of excessive vitamin A supplementation in pwCF with EPI post ETI, especially if exocrine pancreatic function is restored. Children starting ETI ideally should have their plasma/serum vitamin A levels measured before ETI initiation and again within three months post-ETI initiation to monitor for hypervitaminosis [109,111]. It is important to note that intracranial hypertension has been reported even in patients with normal vitamin A levels; therefore, observing an upward trend (rapid increase) may be more clinically relevant [109,148,149,150,151,152]. Similarly, medications such as tetracyclines may act synergistically with vitamin A to induce intracranial hypertension, and this risk could be much higher in pwCF on ETI.

#### 6.1.3. Potential Mechanisms Underlying the Changes in Vitamin A Post-Modulators

Various mechanisms have been proposed to explain the improvement in vitamin A levels after modulators, such as the favorable effects of ETI therapy on the improvement in gastrointestinal milieu, which could increase vitamin A absorption [108,110,142]. Significant reduction in the number of acute pulmonary exacerbations and improvement of the chronic inflammatory status (noted by a decrease in CRP in some studies) with reduced requirement of vitamin A could also increase vitamin A levels [104,105,110,143]. Another reason for improving vitamin A status could be due to improvement in exocrine pancreatic function in the younger population [106,111,153]. However, the changes in vitamin A were also reported irrespective of pancreatic function [110]. On the contrary, there are a few studies that also did not reveal an increase in vitamin A post-modulators [154,155].

The reason for discrepancies in changes in vitamin A could be explained by the following confounding factors: most are retrospective in methodology, age of the patients, changes in vitamin A supplements, and a smaller sample size. Prospective studies with larger samples will help elucidate these changes more precisely.

#### 6.1.4. Current Treatment and Monitoring Recommendations

In the absence of robust evidence, checking vitamin A levels within a few months of starting ETI is recommended to ensure the levels are not supratherapeutic [107,109,110]. Therefore, pwCF on ETI should be closely monitored for vitamin A intake (both from diet as well as from over-the-counter supplements) and symptoms of hypervitaminosis A, such as headache, vomiting, visual changes, lethargy, etc. (detailed in an earlier section) [78]. Current CF-specific vitamins predominantly have provitamin A carotenoids (β-carotene) aimed at minimizing the risk of hypervitaminosis A [109,156,157,158,159,160]. If vitamin A levels reach toxic levels, supplementation should be discontinued immediately with frequent rechecking of the levels.

### 6.2. Pregnancy

With major advancements in CF care including modulator therapy, an increasing number of pwCF are pursuing pregnancy [161]. Studies specific to the management of micronutrition for pwCF, either for those considering pregnancy or during pregnancy, remain elusive, and most recommendations are based on expert guidance [162]. Pregnancy poses unique challenges regarding vitamin A supplementation. Both deficiency and excessive intake should be avoided as vitamin A can be teratogenic in both these circumstances, particularly during early gestation [20,89,96]. Pregnant pwCF may have low body weight due to EPI, and pregnancy’s increased caloric demands may necessitate more aggressive nutritional intervention to decrease the elevated risk of preterm delivery and low birth weight [121]. In utero, the fetus has a high demand for vitamin A, which could predispose it to deficiency [97].

Severe vitamin A deficiency may impair the fetal development of skeletal muscle, heart, respiratory, and nervous systems, contributing to increased risk of preterm birth, low birth weight, and physical defects [163]. On the contrary, doses higher than 10,000 IU (3000 μg) daily in pregnant women can be teratogenic and have been associated with increased risk of miscarriages and also congenital defects [120,122,123,124,125,126]. Vitamin A supplementation (from all sources such as diet, CF-specific vitamins, over-the-counter prenatal vitamins, and oral/enteral supplements) should continue below 10,000 IU/day throughout the pregnancy [4,120,121,164]. As the current CF-specific vitamins have predominantly β-carotene (Supplementary Table S1), toxicity has been less common under close monitoring [121,163].

Ideally, fat-soluble vitamin levels should be tested before conception and monitored every trimester to ensure they are optimized [20,29]. Adjustments should be made using foods and dietary supplements, and retinol levels should be rechecked following changes to supplemental vitamin prescription [162]. To minimize vitamin A toxicity during pregnancy, a standard CF-specific vitamin should be taken every other day, alternating with concurrent use of a prenatal vitamin [161]. In the recent CFF position paper, vitamin A dosage recommendations were explicitly provided [165]. Vitamin A from all sources was recommended at 2300 IU/day for those planning for pregnancy. During pregnancy, a dose of 2500–2600 IU/day was recommended, and additionally individualized doses based on serum vitamin A level (total dose < 10,000 IU/day) were advised. If breastfeeding, the recommended vitamin A dose was 4000–4300 IU/day for the mother [165].

### 6.3. Exocrine Pancreatic Insufficiency

EPI is the most prominent reason for vitamin A deficiency in pwCF, and the risk is higher in pwCF whose malabsorption is not optimally managed and/or pwCF not on CF-specific vitamin supplementation [3,6,12,17,18,85,129]. Further, comorbid clinical instances such as inflammatory bowel conditions (celiac disease and inflammatory bowel disease), or short gut syndrome (intestinal resection due to a history of complicated meconium ileus) could further predispose to reduced absorption of vitamin A [12,17,19,20,85]. Pancreatic-sufficient pwCF are generally at very low risk of vitamin deficiency [131,166].

### 6.4. Advanced Cystic Fibrosis-Related Liver Disease

Estimating vitamin A status in chronic liver disease is challenging, as pwCF are prone to both deficiency and toxicity. Vitamin A deficiency can occur from decreased dietary intake [6,84,94]. Particularly, in aCFLD, people are at risk of vitamin A deficiency due to impaired bile production and flow, which interferes with its absorption. Further, in chronic liver disease, hepatic stellate cells lose lipid droplets containing retinol, and this depletion of storage can predispose to systemic vitamin A inadequacy [50]. The Cystic Fibrosis Foundation (CFF) guidelines recommended a bi-annual nutritional assessment with monitoring of serum fat-soluble vitamin levels and liver function tests, with a close management plan with the input from a registered dietitian and gastroenterologist/hepatologist [92,95]. Supplementing with water-soluble vitamin A (as β-carotene) has been suggested when serum levels are suboptimal [167]. Generally, large doses of vitamin A are required to produce toxicity. However, in people with aCFLD, vitamin A toxicity can develop with relatively lower doses due to limited storage of stellate cells [8,27,74,117]. Excessive vitamin A may activate the stellate cells to transform into myofibroblastic cells, which could lead to the production of excess extracellular matrix, resulting in liver fibrosis [50,168,169]. Given the narrow therapeutic range of vitamin A in people with aCFLD, adjunct evaluation of the functional status of vitamin A could be useful, and referral to an ophthalmologist specifically to evaluate for vitamin A deficiency manifestations can be made.

### 6.5. Post-Lung Transplantation

Significant increase in vitamin A and vitamin E levels after lung transplantation has been noted, and routine careful monitoring is recommended [4,127,128]. When indicated, vitamin A supplementation from diet and supplements should be decreased or totally discontinued, respectively [128]. In some instances, this high vitamin A level occurred even after cessation of the supplementation [4,127,128]. Venu et al. noted that post-transplant vitamin A levels increased in pwCF receiving a transplant between 2004 and 2009 (1.2 ± 0.5 to 3.3 ± 1.4 μmol/L, *p* < 0.0001) [128]. Renal dysfunction may occur due to immunosuppressive medications, and this may result in increased vitamin A levels [91,128]. Stephenson et al. reported increased vitamin A levels in 23 adults with CF who had undergone a bilateral lung transplant and did not have impaired renal function [127]. As the exact etiology of this increase remains elusive, it may be multifactorial and due to altered absorption, reduced inflammatory state, drug interactions, decreased renal clearance, impaired retinol interactions, or increased hepatic synthesis of RBP [170].

### 6.6. Renal Dysfunction

Given the paucity of CF-specific studies evaluating the association of renal insult and vitamin A homeostasis, literature from the non-CF population can be extrapolated. Serum vitamin A levels are often increased in patients with advanced kidney disease, with reduced glomerular filtration. A study of 759 patients with chronic renal disease demonstrated significantly elevated plasma vitamin A levels, and the levels inversely correlated with glomerular filtration rate [118]. Further, in chronic renal disease, serum retinol and RBP are elevated, likely due to increased hepatic RBP synthesis and/or hepatic secretion of RBP-retinol [48,119]. Jing et al. noted that the elevated retinol levels of circulating retinol and its carrier proteins in chronic renal disease were independent of vitamin A intake [48]. Hence, vitamin A and RBP levels should be closely monitored in pwCF with renal insufficiency.

### 6.7. Intestinal Disorders

In people with short bowel syndrome secondary to profound intestinal resection, vitamin A deficiency is more common and should be carefully monitored [19,171,172]. Night blindness and central scotoma in pwCF with short bowel syndrome have been reported [171,172]. Data from non-CF studies have noted increased vitamin A deficiency in people with inflammatory bowel disease and celiac disease [173,174,175,176]. As pwCF are at increased risk of development of these mucosal inflammatory conditions, vitamin A deficiency should be proactively monitored along with other micronutrient deficiencies [177,178,179].

## 7. Vitamin A Dosing

The recommended doses of vitamin A are based on age (Table 3), supplement types (retinol vs. β-carotene), and underlying physiological (e.g., pregnancy) and pathological disease statuses (e.g., advanced liver disease and renal insufficiency). Vitamin A dosing recommendations are mostly based on expert consensus and ULs known in the control population. Regular monitoring and, if needed, individualization of vitamin A supplementation were recommended to prevent both deficiency and excess states.

In severe vitamin A deficiency states involving ophthalmic emergencies, high-dose vitamin A (either oral or intramuscular) can be given [98]. The World Health Organization (WHO) recommends 50,000 IU for infants < 6 months; 100,000 IU for children 6–12 months of age; and 200,000 IU for children over 12 months of age [100,181]. First and second doses are given on consecutive days, and the third dose is given two weeks later [99]. In pregnant patients, high-dose vitamin A can be teratogenic and should be avoided. Intramuscular formulations may not be available on short notice [100]. An interdisciplinary team consisting of a CF physician, a nutrition-focused physician, a registered dietitian, a pharmacist, a registered nurse, a social worker, and an ophthalmologist will be helpful in these circumstances.

## 8. Conclusions

With the changing landscape of nutritional approaches and interventions in the current era of post-CFTR-directed therapies, vitamin A sufficiency status is more often encountered than deficiency. However, there are select circumstances where both vitamin A deficiency and excess (sometimes in the toxic ranges) are not uncommon, and clinicians should be vigilant of both these conditions. We emphasize the need for a thorough review of dietary intake and medication history and ongoing monitoring of fat-soluble vitamin levels and adherence to supplementation. An interdisciplinary approach by a registered dietitian, gastroenterologist, CF physician, clinical pharmacist, social worker, and registered nurse, along with input from other specialists (e.g., ophthalmologist, hepatologist, nephrologist, obstetrician, transplant team, and bone specialist, etc.), is strongly advocated in states of extremely altered vitamin A homeostasis. The current practice for vitamin A is almost solely based on clinical guidance and expert consensus. Similarly, pertinent research from the non-CF population could also be carefully applied to CF-specific circumstances. Adequately powered, multicentered interventional trials are urgently needed to evaluate the role and details (optimal dose, formulation, individualization, etc.) of vitamin A supplementation. Similarly, better markers of vitamin A status are needed, as both serum retinol and RBP are acute-phase reactants and often their measurements are confounded by inflammatory status. Till then, predominantly β-carotene-containing vitamin preparations should be used to minimize the risk of hypervitaminosis A as recommended by most CF guidelines.

## Figures and Tables

**Table 1:** Laboratory parameters (biomarkers) utilized in both deficiency and excess vitamin A status [20,27,53,57,62,63,68,69,70,79,80].

**Vitamin A deficiency**	**Laboratory parameters (biomarkers) recommended**
Serum retinol	Low
RBP	Low
Retinol:RBP molar ratio	<0.8
CRP	May be high in inflammatory conditions
Plasma zinc	Zinc deficiency should be evaluated in refractory vitamin A deficiency
Other fat-soluble vitamin levels	Could be low if decreased intake or malabsorption is the underlying etiology
Miscellaneous tests	Liver function tests and complete blood count may be abnormal in aCFLD
**Vitamin A excess states**	**Laboratory parameters (biomarkers) recommended**
Retinyl esters	>10% of serum retinol A
Serum retinol	High
Retinol:RBP molar ratio	>1.2 (some recommend >1)
Miscellaneous tests	Liver function tests/complete blood count and renal function tests may be abnormal in aCFLD and renal insufficiency, respectively

aCFLD—advanced cystic fibrosis liver disease; CRP—C-reactive protein; RBP—retinol-binding protein.

**Table 2:** Vitamin A deficiency and excess states in cystic fibrosis.

Vitamin A Deficiency States	Possible Etiological Factors	Management Considerations
Exocrine pancreatic insufficiency [16,19,24,91,92,93]	- Suboptimally controlled exocrine pancreatic insufficiency - Patients not adherent to PERT and/or CF-specific vitamins	- Patient education on medications (PERT, CF-specific vitamins) adherence - Optimize dose of PERT and CF-specific vitamins and reevaluate vitamin A levels in 3 months after dose adjustment
Inflammatory states (pulmonary exacerbations and other infection/inflammatory conditions) [12,17,19,20,85]	- Low serum retinol and RBP as both are negative acute phase reactants - High CRP could be noted as well.	- Ideally vitamin A levels should not be obtained during periods of infections or inflammatory states. - Repeat serum retinol and RBP values in 3 months to ensure levels normalized
Advanced CF-associated liver disease [6,84,92,94,95]	- Decreased intake, reduced absorption from the gut, due to impaired bile production and flow, and decreased storage	- Bi-annual nutritional assessment with monitoring of serum fat-soluble vitamin levels and liver function tests - Molar ratio of retinol/RBP should be kept between 0.8 and 1
Pregnancy [20,89,96,97]	- Additionally increased vitamin A need for the developing fetus - Vitamin A deficiency is teratogenic	- Ideally, fat-soluble vitamin levels should be tested before conception and monitored every trimester to ensure they are within normal limits
Short bowel syndrome and inflammatory bowel conditions (e.g., celiac disease and inflammatory bowel disease) [16,19,24,92]	- Reduced vitamin A absorption from the gut - Low vitamin A levels could be related to gut inflammation	- Frequent monitoring of vitamin A levels and dose should be optimized. - Management of underlying condition may enhance gut absorption of vitamin A - In severe cases, parenteral vitamin A may be needed in short bowel syndrome
Further, in people without CF, vitamin A deficiency has also been noted in people with autism, chronic alcoholism, restricted diet, bariatric surgeries, and malnutrition in resource-rich countries [98,99,100,101,102]	- Reduced vitamin A dietary intake	- Frequent monitoring of vitamin A levels and dose should be optimized.
**Vitamin A Excessive States**	**Possible Etiological Factors**	**Management Considerations**
Modulator use [78,103,104,105,106,107,108,109,110,111,112,113,114,115,116]	- Exact etiology unclear and reduced inflammatory state and improved gastrointestinal absorption may be reasons	- Checking vitamin A levels within a few months of starting ETI is recommended to ensure the levels are not supratherapeutic
Advanced CF-associated liver disease [8,20,27,68,69,74,117]	- Reduced storage capacity in the Ito cells and subsequent liver cell injury	- Molar ratio of retinol/RBP should be kept <1.2 (some advocate a ratio of <1)
Chronic renal insufficiency [48,118,119]	- Possible reasons include reduced glomerular filtration of retinol-RBP-TTR complex and increased hepatic RBP synthesis	- Frequent monitoring for vitamin A toxicity should be ensured - Vitamin A supplementation, from diet and supplements, should be decreased or totally discontinued, respectively
Pregnancy [4,120,121,122,123,124,125,126]	- Excess vitamin A is teratogenic and intake should be carefully monitored	- Frequent monitoring for vitamin A toxicity should be ensured - Total vitamin A intake should be <10,000 IU/day throughout the pregnancy
Post lung transplantation [4,127,128]	- Possible etiological factors include altered absorption, reduced inflammatory state, drug interactions, decreased renal clearance, impaired retinol interactions, or increased hepatic synthesis of RBP	- Vitamin A supplementation, from diet and supplements, should be decreased or totally discontinued, respectively. - Frequent monitoring for vitamin A toxicity should be ensured
High vitamin A intake [20,90]	- Medications such as isotretinoin and acitretin and over-the-counter vitamins (including water-soluble vitamin A preparations) may increase the total intake of vitamin A	- Reduce the dose and ensure levels are optimized

CF—cystic fibrosis, CRP—C-reactive protein, IU—international units; PERT—pancreatic enzyme replacement therapy; RBP—retinol-binding protein, TTR—transthyretin.

**Table 3:** Vitamin A dose recommendations by various global cystic fibrosis organizations.

Daily Vitamin A Recommendation	Dose (IU)
The CFF recommendation [30]	<1 year—1500 IU 1–3 years—5000 IU 4–8 years—5000 to 10,000 IU >8 years—10,000 IU
Australia and New Zealand CF guidelines [20,180]	<1 year—1500 to 2000 IU Young children—1500 to 5000 IU Older children—2500 to 5000 IU Teens and adults—2500 to 5000 IU
European guidelines [4,28,29]	Initial dose—β-carotene at a dose of 1 mg/kg/day (maximum 50 mg/day) for 12 weeks and followed by maintenance dose (maximum of 10 mg/day)
Canadian (British Columbia) guidelines [27]	Similar to the European guidelines regarding β-carotene dosage For retinol, initiate at a low dose and titrate up to the target range
Brazilian guidelines [26]	<1 year—1500 IU 1–3 years—5000 IU 4–8 years—5000 to 10,000 IU >8 years—10,000 IU

CF—cystic fibrosis; CFF—Cystic Fibrosis Foundation; IU—international units.

## Data Availability

No new data were created or analyzed in this study. Data sharing is not applicable to this article.

## Supplementary Materials

The following supporting information can be downloaded at: https://www.mdpi.com/article/10.3390/nu18162639/s1, Table S1: Commercial products with vitamin A available specifically for people with cystic fibrosis [40,41,42,43].

## Abbreviations

aCFLD—advanced cystic fibrosis liver disease; CF—cystic fibrosis; CFF—Cystic Fibrosis Foundation; CFTR—cystic fibrosis transmembrane-conductance regulator; CRP—C-reactive protein; CYP26—cytochrome P450 family 26; EPI—exocrine pancreatic insufficiency; ETI—elexacaftor/tezacaftor/ivacaftor; IU—international units; NHANES—National Health and Nutrition Examination Survey; PERT—pancreatic enzyme replacement therapy; pwCF—people with cystic fibrosis; RAE—retinol activity equivalent; RBP—retinol-binding protein; SBS—short bowel syndrome; STRA6—stimulated by retinoic acid 6; TTR—transthyretin; UL—tolerable upper intake level; WHO—World Health Organization.
