# Supplementary material for: Vitamin A Status in Cystic Fibrosis in the Current Era of CFTR-Directed Therapies
Source: Nutrients. 2026 Aug 12;18(16):2639. doi: 10.3390/nu18162639 (PMC13516817; doi:10.3390/nu18162639)
Supplement: Supplementary file 1 [file nutrients-18-02639-s001.zip › nutrients-4437653-supplementary.pdf]

Supplementary Table S1. - Commercial products with vitamin A available specifically for people with cystic fibrosis [40-43].

**Products available in the market[#]**	**Total vitamin A dosage (retinol and β-carotene)**
DEKAs Plus Drops®	5,751 IU (1,725 mcg RAE)/ one mL (87% as β-carotene)
DEKAs Essential Capsules®	2,000 IU (606 mcg RAE)/ one capsule (66% as β-carotene)
DEKAs Plus Chewables®	18,167 IU (5,450 mcg RAE) /one chewable (92% as β-carotene)
DEKAs Plus Softgels®	18,167 IU (5,450 mcg RAE)/one softgel (92% as β-carotene)
MVW Complete Formulation® Drops	9,254 IU (2,777 mcg RAE)/ one mL (75% as β-carotene)
MVW Complete Formulation® Chewables	16,000 IU (4,800 mcg RAE)/ one chewable (88% as β-carotene)
MVW Complete Formulation® ADEK Gummies	16,000 IU (4,800 mcg RAE)/ two gummies (88% as β-carotene)
MVW Complete Formulation® Softgels /Softgel Minis	32,000 IU (9,600 mcg RAE)/ two softgels or four minis (88% as β-carotene)
MVW Modulator Formulation® Drops	3,566 IU (2,000 mcg RAE)/ three mL (93% as β-carotene)
MVW Modulator Formulation® Chewables	11,730 IU (6,800 mcg RAE)/ two chewables (96.5% as β-carotene)
MVW Modulator Formulation® Softgel Minis	40,000 IU (12,000 mcg RAE)/ four softgel minis (96.5% as β-carotene)

(# Products grouped under alphabetical order, and please refer to the manufacturers' product information for the latest updates)
